# Retraction Note: β_2_-adrenoreceptor Inverse Agonist Down-regulates Muscarine Cholinergic Subtype-3 Receptor and Its Downstream Signal Pathways in Airway Smooth Muscle Cells *in vitro*

**DOI:** 10.1038/s41598-019-39065-w

**Published:** 2019-03-06

**Authors:** Jian Luo, Yuan-hua Liu, Wei Luo, Zhu Luo, Chun-tao Liu

**Affiliations:** 1 Department of Respiratory Diseases, West China School of Medicine and West China Hospital, Sichuan University, Chengdu, 610041 China; 2 Department of Respiratory Diseases, The First Affiliated Hospital, Zhengzhou University, Zhengzhou, 450052 China; 3 Department of Respiratory Diseases, The First Affiliated Hospital, Chongqing Medical University, Chongqing, 400016 China

Retraction of: *Scientific Reports* 10.1038/srep39905, published online 04 January 2017

The Authors are retracting this Article.

During the preparation of the figures the Authors included previously published microscopy and Western blotting data in Figures 1, 2, 3, and 7. Additionally, the authors are unable to locate the correct original data for the Western blot experiments and therefore cannot guarantee the accuracy of the data presented in Figures 2, 3, and 7, which is key to the conclusions of this Article.

All authors agree to the retraction.

